# Decreased NSD2 impairs stromal cell proliferation in human endometrium via reprogramming H3K36me2

**DOI:** 10.1530/REP-23-0254

**Published:** 2024-02-12

**Authors:** Chuan-Mei Qin, Xiao-Wei Wei, Jia-Yi Wu, Xue-Qing Liu, Yi Lin

**Affiliations:** 1 The International Peace Maternity and Child Health Hospital, School of Medicine, Shanghai Jiao Tong University, Shanghai, China; 2 Shanghai Key Laboratory of Embryo Original Diseases, Shanghai, China; 3 Institute of Birth Defects and Rare Diseases, School of Medicine, Shanghai Jiao Tong University, Shanghai, China; 4 Shanghai Jiao Tong University School of Medicine Affiliated Sixth People's Hospital, School of Medicine, Shanghai Jiao Tong University, Shanghai, China

## Abstract

**In brief:**

The proliferation of the endometrium is regulated by histone methylation. This study shows that decreased NSD2 impairs proliferative-phase endometrial stromal cell proliferation in patients with recurrent implantation failure via epigenetic reprogramming of H3K36me2 methylation on the promoter region of *MCM7*.

**Abstract:**

Recurrent implantation failure (RIF) is a formidable challenge in assisted reproductive technology because of its unclear molecular mechanism. Impaired human endometrial stromal cell (HESC) proliferation disrupts the rhythm of the menstrual cycle, resulting in devastating disorders between the embryo and the endometrium. The molecular function of histone methylation enzymes in modulating HESC proliferation remains largely uncharacterized. Herein, we found that the levels of histone methyltransferase nuclear receptor binding SET domain protein 2 (NSD2) and the dimethylation of lysine 36 on histone H3 are decreased significantly in the proliferative-phase endometrium of patients with RIF. Knockdown of* NSD2* in an HESC cell line markedly impaired cell proliferation and globally reduced H3K36me2 binding to chromatin, leading to altered expression of many genes. Transcriptomic analyses revealed that cell cycle-related gene sets were downregulated in the endometrium of patients with RIF and in *NSD2*‑knockdown HESCs. Furthermore, RNA-sequencing and CUT&Tag sequencing analysis suggested that *NSD2* knockdown reduced the binding of H3K36me2 to the promoter region of cell cycle marker gene *MCM7* (encoding minichromosome maintenance complex component 7) and downregulated its expression. The interaction of H3K36me2 with the *MCM7* promoter was verified using chromatin immunoprecipitation–quantitative real-time PCR. Our results demonstrated a unifying epigenome-scale mechanism by which decreased NSD2 impairs endometrial stromal cell proliferation in the proliferative-phase endometrium of patients with RIF.

## Introduction

Recurrent implantation failure (RIF), defined as two or more implantation failures for individuals, occurs in about 10% of infertile women undergoing *in vitro* fertilization–embryo transfer (IVF-ET) (Coughlan *et al.* 2014, Kliman & Frankfurter 2019, Garneau & Young 2021). Failed embryo implantation can be caused by impaired embryo quality, a non-receptive endometrium, or abnormal crosstalk between the two (Zhang *et al.* 2013). Two-thirds of implantation failures are ascribed to inadequate uterine receptivity (Munro *et al.* 2010), which is associated with dysfunctional proliferation and differentiation of endometrial stromal cells (Woods *et al.* 2017).

Endometrial stromal cells, endometrial epithelial cells, and immune cells mainly form the functional layer of the endometrium. The endometrium undergoes cyclical proliferation, differentiation, and shedding throughout the menstrual cycle (Santamaria *et al.* 2018). Primary human endometrial stromal cells from the early proliferative-phase endometrium of patients with implantation failure display markedly impaired decidualization (Mackens *et al.* 2020). Correct endometrial proliferation during the proliferative phase is vital for embryo implantation (Zhao *et al.* 2022).

Proliferating stromal cells exhibit cell–cell interaction hubs during the rapid growth of the proliferative-phase endometrium (Fang *et al.* 2022). Epigenetic regulation of proliferation increases the acquired estrogen receptors in endometrial stromal cells via a high level of methylated cytosines (Munro *et al.* 2010). Furthermore, endometrial epithelial cell proliferation is indirectly regulated by paracrine factors in endometrial stromal cells upon estrogen stimulation through H3K4me1/3, H3K9ac, and H3K27ac (Tong *et al.* 2019). Epigenetics refers to reversible DNA modifications and the histone proteins that alter gene expression without changing its DNA sequence (Biel *et al.* 2005). Histone posttranslational modifications (methylation, acetylation, phosphorylation, and ubiquitination), dynamically modulate chromatin structure and function, thereby regulating gene expression (Millán-Zambrano *et al.* 2022). In the early 1990s, it was proposed that histone gene transcription was enhanced during the early S phase of the cell cycle (Stein *et al.* 1994). Histone methylation is a stable and heritable epigenetic histone posttranslational modification, mainly occurring on lysine and arginine residues of histones H3 and H4 (Al Ojaimi *et al.* 2022). During the cell cycle, H3K9me3, H3K27me3, and H3K36me2 delimit the majority of the human genome into mutually unshared regions (Van Rechem *et al.* 2021). H3K9me3 gradually increases through G1 to S phase, then decreases sharply between S and G2 phases to moderate the synthesis of new DNA copies (Stewart-Morgan *et al.* 2020). H3K27me3 regions occurring throughout S phase moderate chromatin accessibility across the cell cycle. H3K36me2 occupies 29% of the genome and is highly conserved. Furthermore, the H3K36me2 state is uncharacterized among primary chromatin states (Van Rechem *et al.* 2021). The histone methyltransferase enhancer of Zeste 2 Polycomb Repressive Complex 2 Subunit (EZH2) regulates endometrial proliferation and differentiation via H3K27me3 (Valatkaitė *et al.* 2021). H3K27me3 coordinates with H3K27ac at promoters to regulate the expression of genes crucial to decidualization, which does not involve H3K9me3 (Katoh *et al.* 2018).

Compared with H3K9me3 and H3K27me3, there are relatively few reports on H3K36me2. At least six histone methyltransferases are capable of catalyzing H3K36me2 formation: nuclear receptor binding SET domain protein 1 (NSD1), NSD2, NSD3, absent small and homeotic disks protein 1 homolog (ASH1L), lysine *N*-methyltransferase 3C (KMT3C), and KMT3A (Greer & Shi 2012). NSD2 is a newly identified histone methylation transferase with specific dimethyltransferase activity on H3K36, generating H3K36me2. NSD2 is essential to maintain chromatin integrity and regulate the expression of genes controlling cell proliferation, apoptosis, and DNA repair (Kuo *et al.* 2011, Topchu *et al.* 2022). NSD2 is a critical molecule in cell senescence (Tanaka *et al.* 2020), T cell differentiation (Long *et al.* 2020), cell proliferation (Chen *et al.* 2020), and tumor metastasis (Chen *et al.* 2019). Whether NSD2 modulates the proliferation of proliferative-phase endometrial stromal cells, and their related epigenetic regulation, is unclear.

The present study focused on the proliferative-phase endometrium of patients with RIF and fertile controls, including screening for genes that regulate histone methylation, and identifying the epigenome-wide mechanisms affecting endometrial stromal cell proliferation.

## Materials and methods

### Patient characteristics

Twelve patients (mean age, 34.08 ± 3.40 years) with RIF and twelve fertile (FER) controls (mean age, 33.55 ± 3.98 years) were recruited from the Department of Obstetrics and Gynecology in the International Peace Maternity & Child Health Hospital from September 2020 to February 2021. Proliferative-phase endometrial samples were collected during diagnostic hysteroscopy procedures. Patients with a history of no pregnancies after at least two embryo transfers were recruited into the RIF group. The FER group comprised patients who previously had successful pregnancies without any complications. Pathology results for all endometrial biopsies were completely normal. All patients were under 40 years old with regular menstrual cycles. Women with hydrosalpinx, endometriosis, or adenomyosis, and patients treated with hormonal therapy within the last 6 months, were excluded. The study protocol was approved by the Medical Ethics Committee of the International Peace Maternity & Child Health Hospital of China Welfare Institute, Shanghai ((GKLW) 2021-49). Written informed consent was obtained from all the participants before enrolment (FER and RIF groups).

### Cell culture

The immortalized human endometrial stromal cell line (HESCs) was a gift from Professor Wang Haibin (ATCC® CRL-4003™) (Jiang *et al.* 2020, Wei *et al.* 2022). The cells were grown in phenol red-free Dulbecco’s modified Eagle’s medium: nutrient mixture F-12 (DMEM/F12, Gibco) with 10% charcoal-stripped fetal bovine serum (CS-FBS, Biological Industries, Beit Haemek, Israel), 100 μg/mL streptomycin, 100 IU/mL penicillin, 1% insulin–transferrin–selenium solution (ThermoFisher Scientific), and 500 ng/mL puromycin (Sigma-Aldrich) at 37°C with 5% CO_2_. The cell culture medium was exchanged every 48 h. For passaging, the cells were detached using trypsin with EDTA (Sigma) at 37°C for 3 min.

### *NSD2* knockdown

HESCs at 30–50% confluence were transfected with a small interfering RNA (siRNA) against *NSD2* or a randomly scrambled siRNA (negative control (NC); GenePharma, Shanghai, China) using Lipofectamine RNAiMAX Transfection Reagent (Invitrogen) following the manufacturer’s instructions. The sequences of siNSD2 are provided in Supplementary Table 1 (see section on supplementary materials given at the end of this article).

The* NSD2* short hairpin RNA (shRNA; CCAGAAAGAGCTTGGATATTT) was subcloned from the pLV vector (VectorBuilder, Guangzhou, China) into the U6 promoter and terminator sites. A scrambled shRNA sequence was used as the NC. HESCs at 30–50% confluence were transfected with the control shRNA virus or shRNA virus (multiplicity of infection (MOI) = 20) with 1 mL cell culture medium per well in a six-well plate. About 16 h later, the medium was discarded and cells were washed once with phosphate-buffered saline (PBS) and then incubated in normal medium.

Cells were collected for RNA and protein analysis after 48 h.

### Lentiviral overexpression

The lentiviral overexpression construct pLV-NSD2 and control pLV vectors were purchased from VectorBuilder and used to infect HESCs at an MOI of 20. An appropriate amount of lentivirus was added to 1 mL of cell culture medium with 10% CS-FBS per well in a six-well plate containing cells at 30–50% confluence. About 16 h later, the virus-containing culture medium was discarded and cells were washed with PBS once. Then, fresh complete culture medium with 10% CS-FBS was added to the cells. To select NSD2 stably expressing cells, culture medium with 75 μg/mL G418 (Invitrogen) was added to cells the day after lentiviral transduction. Cells were selected with G418 for 7 days and then cultured in 10% CS-FBS medium.

### Western blotting

Whole cells or human endometrial tissues were lysed using radioimmunoprecipitation assay buffer (Thermo Fisher Scientific) including a protease inhibitor cocktail (ThermoFisher Scientific). About 20 μg protein extract were separated using sodium dodecyl sulfate–polyacrylamide gel electrophoresis and transferred to a polyvinylidene fluoride membrane. The membranes were blocked in 5% skimmed milk at room temperature for 1 h and then incubated with primary antibodies against NSD2 (1:1000; #65127, Cell Signaling Technology), H3K9me3 (1:1000, ab176916, Abcam), H3K27me3 (1:1000, ab192985, Abcam), H3K36me1 (1:1000, ab176920, Abcam), H3K36me2 (1:1000, ab176921, Abcam), H3K36me3 (1:1000, ab176916, Abcam), and β-actin (ACTB; 1:10,000, 66009-1-Ig, Proteintech, Rosemont, IL, USA) at 4°C overnight. Next day, the membranes were incubated with horseradish peroxidase (HRP)-conjugated secondary antibodies at room temperature for 1 h. The membranes were visualized using Chemiluminescent HRP Substrate (Millipore). The immunoreactive protein band density was quantified using Image J software (NIH). Raw data of all western blots are provided in Supplementary Fig. 4.

### Immunohistochemical staining

Proliferative-phase endometrial tissues from 10 patients with RIF and 10 FER controls were collected and washed with PBS immediately. Tissues were fixed in 4% paraformaldehyde, paraffin-embedded, and serially sectioned. Immunostaining was performed according to the instruction of the mouse and rabbit specific HRP/diaminobenzidine (DAB) (ABC) Detection IHC Kit (ab64264, Abcam). The slides were incubated with primary antibody against NSD2 (1:100, ab223694, Abcam), H3K36me2 (1:1000, ab176921, Abcam), minichromosome maintenance complex component 7 (MCM7) (1:500, 11225-1-AP, Proteintech), or Rabbit IgG (1:500, #3900, Cell Signaling Technology) diluted in PBS overnight at 4°C. The primary antibodies used in the study were all commercial antibodies reported by other empirical studies (Zhu *et al.* 2019, Dai *et al.* 2020, Ganig *et al.* 2021, Acke *et al.* 2022). For each immunohistochemical run, a slice from each group was randomly selected as the negative control. The negative control was tested by incubation with immunoglobulin G (IgG) from the corresponding species. Next day, they were incubated with secondary antibodies, stained with DAB, and counterstained with hematoxylin. The slides were then dehydrated, cleared, and mounted. The images were captured under a microscope. See Supplementary methods for the immunohistochemical scoring method.

### RNA isolation and qRT-PCR

Cells were lysed in TRIzol reagent (Life Technologies) to extract total RNA. After RNA quantification, about 1 μg total RNA was reverse transcribed into cDNA using a PrimeScript RT reagent kit (Takara). Quantitative real-time PCR (qPCR) was performed using a SYBR Green Kit (Takara) with the primers listed in Supplementary Table 2. The housekeeping gene *ACTB* was used as an internal control. See Supplementary methods for the statistical methods used to analyze the qRT-PCR data.

### EdU assay

At about 48 h after transfection, cells were incubated with 50 μM 5′-ethynyl-2′-deoxyuridine (Cell-Light EdU Apollo 643 In Vitro Kit, RiboBio, Guangzhou, China) for 2 h at 37°C and fixed with 4% paraformaldehyde. The cells were then permeabilized using 0.5% Triton X-100 and fluorescently stained according to the manufacturer’s instructions.

### Cell counting Kit-8 assay

About 2500 cells/per well were plated in 96-well plates, with three replicates in each group. One day later, the cells were infected with the corresponding virus at an MOI = 20. Optical density (OD) values at 450 nm were assessed using CCK8 (Yeasen, Shanghai, China) at 0, 24, 48, 72, and 96 h.

### Cell cycle flow cytometry analysis

At 48 h after lentivirus transfection, cells were digested with trypsin and washed twice with PBS. About 5 × 10^5^ cells were collected and fixed in 70% ethanol at 4°C overnight. Then, the cells were centrifuged at 1000 ***g*** for 5 min and resuspended in 500 μL of 1× staining buffer containing 10 μL of propidium oxide and 10 μL of RNase A (Yeasen) for 30 min at 37°C in the dark and then subjected to flow cytometry analysis. The cell cycle status was evaluated using ModFit software (Verity Software House, Topsham, ME, USA).

### RNA sequencing and data analysis

For RNA extraction from human endometrium tissues, tissues were rinsed with PBS and ground in 1 mL of TRIzol (Invitrogen). HESCs were transfected with siNC or siNSD2 for 48 h. Total RNA from the two groups was extracted using 1 mL of TRIzol. Transcriptome sequencing and analysis were conducted by Novogene (Beijing, China). Briefly, NEBNext® Ultra™ RNA Library Prep Kit for Illumina® (NEB, Ipswich, MA, USA) was used to construct the sequencing libraries. After qualification, the libraries were sequenced using the Illumina NovaSeq 6000 system (Illumina, San Diego, CA, USA). DESeq2 R package was used for differential expression analysis between two groups of human endometrium tissues (Love *et al.* 2014). Differential gene expression analysis of HESCs RNA-seq data was performed using edgeR (Robinson *et al.* 2010). Gene Ontology (GO) analysis, Kyoto Encyclopedia of Genes and Genomes (KEGG) analysis, and Gene Set Enrichment Analysis (GSEA) of differentially expressed genes were performed using clusterProfiler R package (3.8.1) (Wu *et al.* 2021). For the HESCs RNA-seq data (*n* = 3), the threshold value for significantly differential expression was an adjusted *P* < 0.05 and |log2foldChange| >1. For the bulk tissue RNA-seq (*n* = 6), the threshold value for significantly differential expression was* P* < 0.05 and |log2foldChange| > 0.75.

### CUT&Tag library generation and sequencing

The CUT&Tag assay was performed in strict accordance with the manufacturer’s instructions (TD903, Vazyme, Nanjing, China). HESCs at 30–50% confluence were plated into 6-well plates and transfected with either control shNC virus or shNSD2 virus (MOI = 20). About 16 h later, the medium was changed to 2 mL of normal medium, and the cells were collected for the CUT&Tag assay after 48 h. Approximately 50,000 HESCs were collected as one sample. Anti-Histone H3 (di methyl K36) antibody (1:50, ab176921, Abcam) was used as the primary antibody. CUT&Tag-Seq and data analysis were then performed by Novogene (Kaya-Okur *et al.* 2019).

### Chromatin immunoprecipitation–quantitative real-time PCR experiments

ChIP was performed using a SimpleChIP^®^ Enzymatic Chromatin IP Kit (9003S, Cell Signaling Technology). For ChIP-qPCR, HESCs at 30–50% confluence were plated on 15 cm culture dishes and transfected with either control shNC virus or shNSD2 virus (MOI = 20). About 16 h later, the medium was changed to 20 mL normal medium, and cells were collected for ChIP experiments after 48 h (~4 × 10^6^ cells for each reaction). ChIP experiments, including sample preparation, nuclei preparation, chromatin digestion, analysis of chromatin digestion and concentration, chromatin immunoprecipitation, elution of chromatin, reversal of cross-links and qPCR were conducted strictly according to the kit instructions (Martin-Martin *et al.* 2016). For H3K36me2 occupied regions, ChIP experiments were performed using anti-histone H3 (di methyl K36) antibodies (2 μg for 25 μg of chromatin, ab176921, Abcam). The DNA products were quantified using qPCR. The ChIP primers were designed using Primer6 software and are listed in Supplementary Table 2. The region of the *MCM7* promoter containing the H3K36me2-binding sites were amplified using a SYBR Green Kit (Takara). Statistical analyses were performed according to the instruction manual: Percent input = 2% × 2^(C[T] 2% Input sample − C[T] IP sample)^.

### Statistical analysis

For data that did not conform to a normal distribution, the Mann–Whitney *U* test was used for comparisons between two groups. For data displaying a normal distribution, Student’s *t*-test or Welch’s *t*-test was used for comparisons between two groups. Comparisons between multiple groups were performed using two-way ANOVA. All *P*-values were two-sided and a value less than 0.05 was regarded as statistically significant. All statistical calculations were performed using SPSS 26 (IBM Corp.). See Supplementary methods for the detailed statistical methods.

## Results

### NSD2 is decreased in the proliferative-phase endometrium of patients with RIF

**Figure 1:**
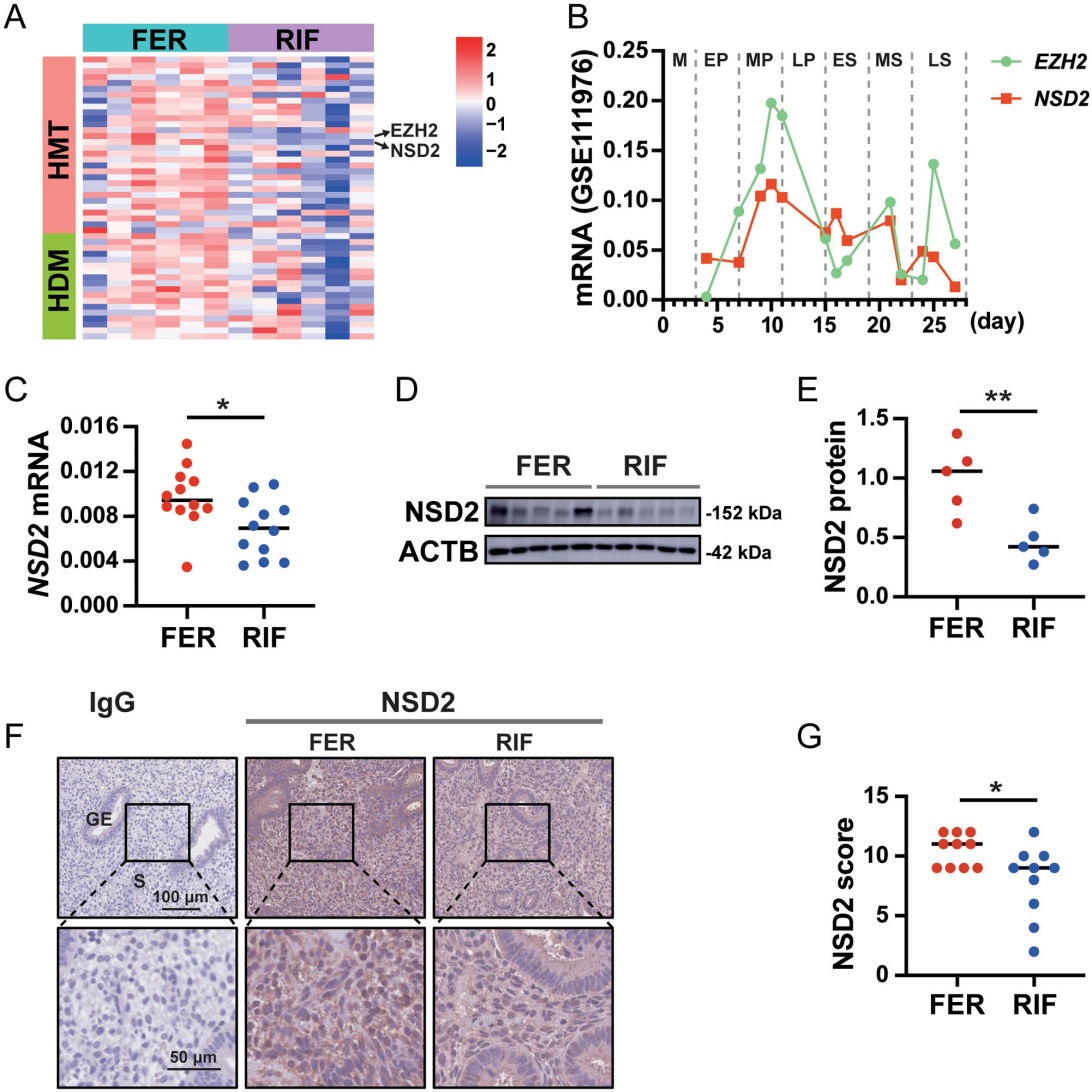
NSD2 expression is decreased in the proliferative-phase endometrium of patients with RIF compared with that of fertile controls (FER). (A) Heatmap of histone methyltransferases (HMT) and histone demethylase (HDM) gene levels in the RIF and FER groups. (B) *EZH2* and *NSD2* expression levels in endometrial stromal cells during the menstrual cycle (GES111976). The median menstrual cycle is 28 days in length. The details of staging in the menstrual cycle are described in Supplementary Table 3. M, menstrual phase; EP, early-proliferative; MP, mid-proliferative; LP, late-proliferative; ES, early-secretory; MS, mid-secretory; LS, late-secretory. (C) *NSD2* mRNA levels in RIF (*n* = 12) and FER (*n* = 12) proliferative endometrium as measured using qRT-PCR. (D, E) Protein levels of NSD2 in the proliferative endometrium of patients with RIF (*n* = 5) and FER controls (*n* = 5) as measured using western blotting. (F, G) Representative immunohistochemical images depicting the expression of NSD2 in the proliferative endometrium from patients with RIF (*n* = 10) and FER controls (*n* = 10). The negative control was normal rabbit IgG. GE, glandular epithelium; S, stroma; *P < 0.05; **P < 0.01.

To evaluate histone methyltransferase/histone demethylase function in proliferative-phase endometria, six endometrial samples from patients with RIF and six from FER controls were subjected to RNA-seq analysis. Studies have proven that many histone methyltransferases and demethylases genes play a role in human health (Greer & Shi 2012). In the RIF group compared with the FER group, the expression levels of 1233 genes were downregulated and 1830 were upregulated (Supplementary Fig. 2A). The results identified *EZH2* and *NSD2* as the primary differentially expressed genes associated with histone methylation (Fig. 1A and Supplementary Fig. 2B). GO and KEGG analyses identified highly significant enrichment of terms for diverse biological processes and diseases, including the cell cycle (Supplementary Fig. 2C). To clarify the expression of EZH2 and NSD2 during the menstrual cycle, we then analyzed published single-cell RNAseq data of the endometrium during human menstrual cycle (Wang *et al.* 2020*b*), which showed that *EZH2* and *NSD2* expression levels gradually increased during the early- and mid-proliferative phases of the menstrual cycle (Fig. 1B). The role of EZH2 in endometrial function has been relatively clear. Throughout the menstrual cycle, the expression of EZH2 in endometrium was significantly lower in secretory phase compared with proliferative phase (Grimaldi *et al.* 2011). In experiments with mice, *Ezh2* deletion enhances endometrial stromal cell senescence and results in pregnancy loss (Sirohi *et al.* 2023). EZH2 was reduced in the endometrium of patients with RIF and the reduction of Ezh2 in the uterine epithelium and stroma of Ezh2-deleted mice exhibited dysregulation of cell cycle regulators and severe subfertility (Fukui *et al.* 2023). The important role of EZH2 in endometrial function suggests reduced NSD2 in the endometrium of patients with RIF may lead to endometrial dysfunction. However, the role of NSD2 in RIF is still unclear. Hence, we focused on the function of NSD2 in our study. qRT-PCR analysis verified that patients with RIF had lower *NSD2* expression in the proliferative-phase endometrium than the FER controls (Fig. 1C), consistent with the western blotting results (Fig. 1D and E). Immunofluorescence staining of HESCs showed that NSD2 was mainly expressed in the nucleus (Supplementary Fig. 3). Immunohistochemical staining showed that NSD2 expression could be detected in both the cytoplasm and the nucleus of both endometrial epithelial and stromal cells and was significantly decreased in endometrial stromal cells from patients with RIF (Fig. 1F and G). Hence, these results suggested that alteration of NSD2 expression in the proliferative-phase endometrium is associated with IVF failure.

### *NSD2* knockdown inhibits human endometrial stromal cell proliferation

**Figure 2:**
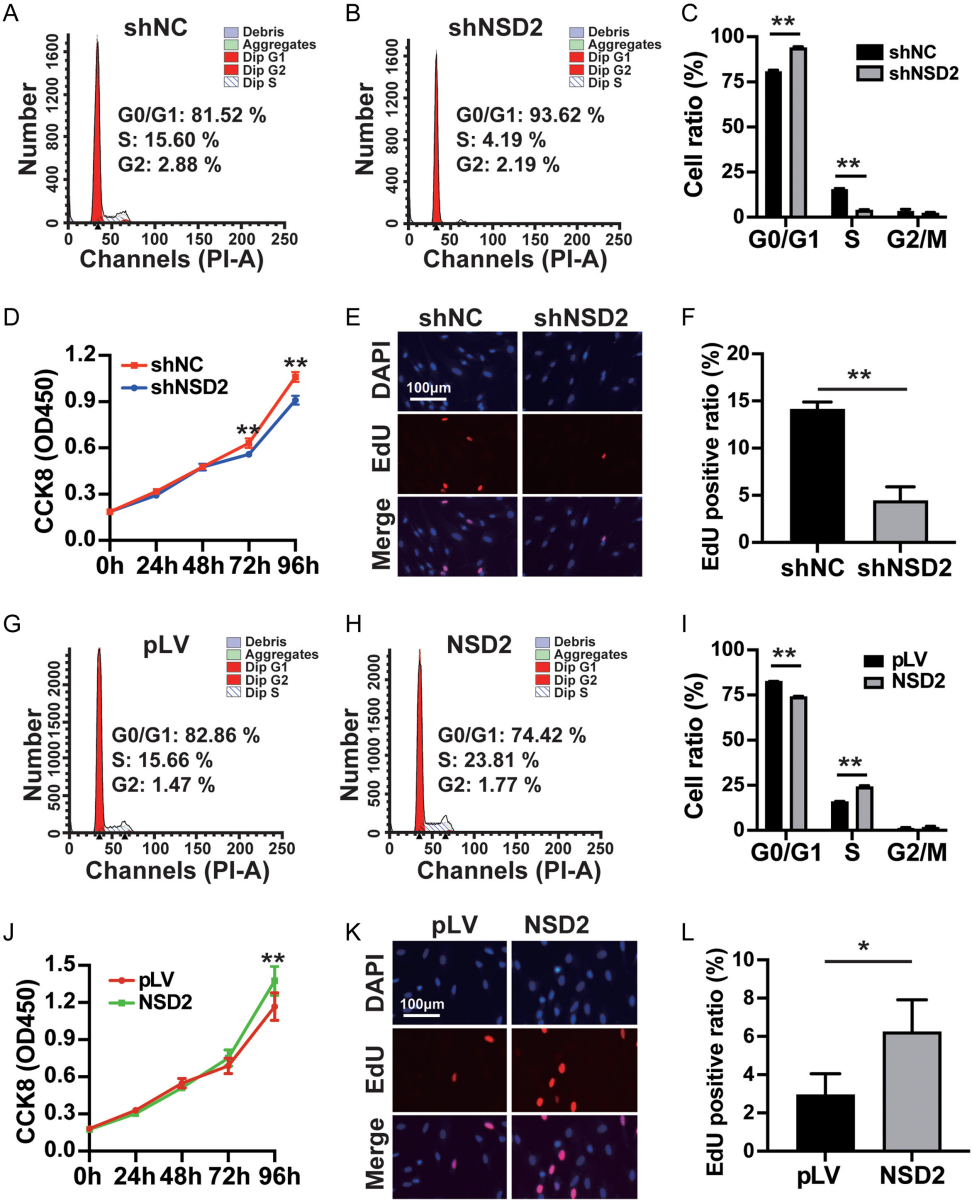
NSD2 regulates the proliferation of HESCs. (A, B, C) Flow cytometry showing the proportion of HESCs in different cell cycle phases in the shNSD2 group compared with that in the shNC group, the mean percentage of HESCs in G0/G1 phase increased from 80.91 ± 0.69% to 93.89 ± 0.63% (*P* < 0.01), the mean percentage of HESCs in S phase decreased from 15.58 ± 0.35% to 3.87 ± 0.32% (*P* < 0.01), and the mean percentage of HESCs in G2/M phase decreased from 3.51 ± 0.96% to 2.24 ± 0.96% (*P* = 0.11). (D) Cell Counting Kit-8 assay showing that decreased NSD2 level impaired HESCs proliferation at 72 h (the mean OD_450_ decreased from 0.54 ± 0.03 to 0.47 ± 0.01, *P* < 0.01) and 96 h (the mean OD_450_ decreased from 0.92 ± 0.06 to 0.78 ± 0.02, *P* < 0.01) compared with that in the shNC group. (E, F) EdU assay showing that the incorporation of the EdU label was diminished in shNSD2 cells (red). The proportion of EdU-positive cells decreased from 14.19 ± 0.70% to 4.48 ± 1.40% after *NSD2* knockdown (*P* < 0.01). (G, H, I) Flow cytometry showing the proportion of HESCs in different cell cycle phases in the *NSD2* overexpressing group compared with that in the pLV group, the mean percentage of HESCs in G0/G1 phase decreased from 82.81 ± 0.08% to 74.18 ± 0.32% (*P* < 0.01), the mean percentage of HESCs in S phase increased from 15.85 ± 0.22% to 24.24 ± 0.55% (*P* < 0.01), and the mean percentage of HESCs in G2/M phase decreased from 1.34 ± 0.14% to 1.57 ± 0.67% (*P* = 0.94). (J) Cell Counting Kit-8 assay showing that *NSD2* overexpression promotes HESC proliferation at 96 h (the mean OD_450_ increased from 1.07 ± 0.11 to 1.28 ± 0.12, *P* < 0.01). (K, L) EdU assay showing that the incorporation of EdU-label increased in *NSD2* overexpressing cells (red). The proportion of EdU-positive cells increased from 2.95 ± 1.10% to 6.24 ± 1.67% after *NSD2* overexpression (*P* = 0.047). Each set of the experiments was replicated at least three times. shNC, pLV-Neo-U6; shNSD2, pLV[shRNA]-Neo-U6>hNSD2; pLV, pLV-Neo-CMV; NSD2, pLV[Exp]-Neo-CMV>hNSD2; *P < 0.05; **P < 0.01.

To explore NSD2's effect on cell proliferation, we constructed a lentivirus-mediated *NSD2*-knockdown cell model. Western blotting and qRT-PCR verified the knockdown efficiency (Supplementary Fig. 1A, B, and C). Further, flow cytometry showed that HESCs were arrested in the G0/G1 stage in the shNSD2 group compared with those in the shNC group (Fig. 2A, B, and C). A CCK8 assay also demonstrated that cell proliferation in the *NSD2* knockdown group was significantly lower than that in the shNC group at 72 h and 96 h (Fig. 2D). The EdU assay also showed that the proportion of EdU-positive cells decreased after *NSD2* knockdown (Fig. 2E and F). Overall, *NSD2* knockdown inhibited HESC proliferation.

### *NSD2* overexpression promotes human endometrial stromal cell proliferation

To further study the role of NSD2 in HESC proliferation regulation, NSD2 overexpression lentivirus was transduced into HESCs. NSD2 overexpression efficiency was verified using qRT-PCR and western blotting (Supplementary Fig. 1D, E, and F). Flow cytometry showed that NSD2 overexpression promoted the G0/G1 to S phase transition (Fig. 2G, H, and I). CCK8 assays revealed increased cell proliferation in the NSD2-overexpression group (Fig. 2J), which was also confirmed using EdU assays (Fig. 2K and L).

### NSD2 knockdown leads to a global decrease in H3K36me2 levels

**Figure 3:**
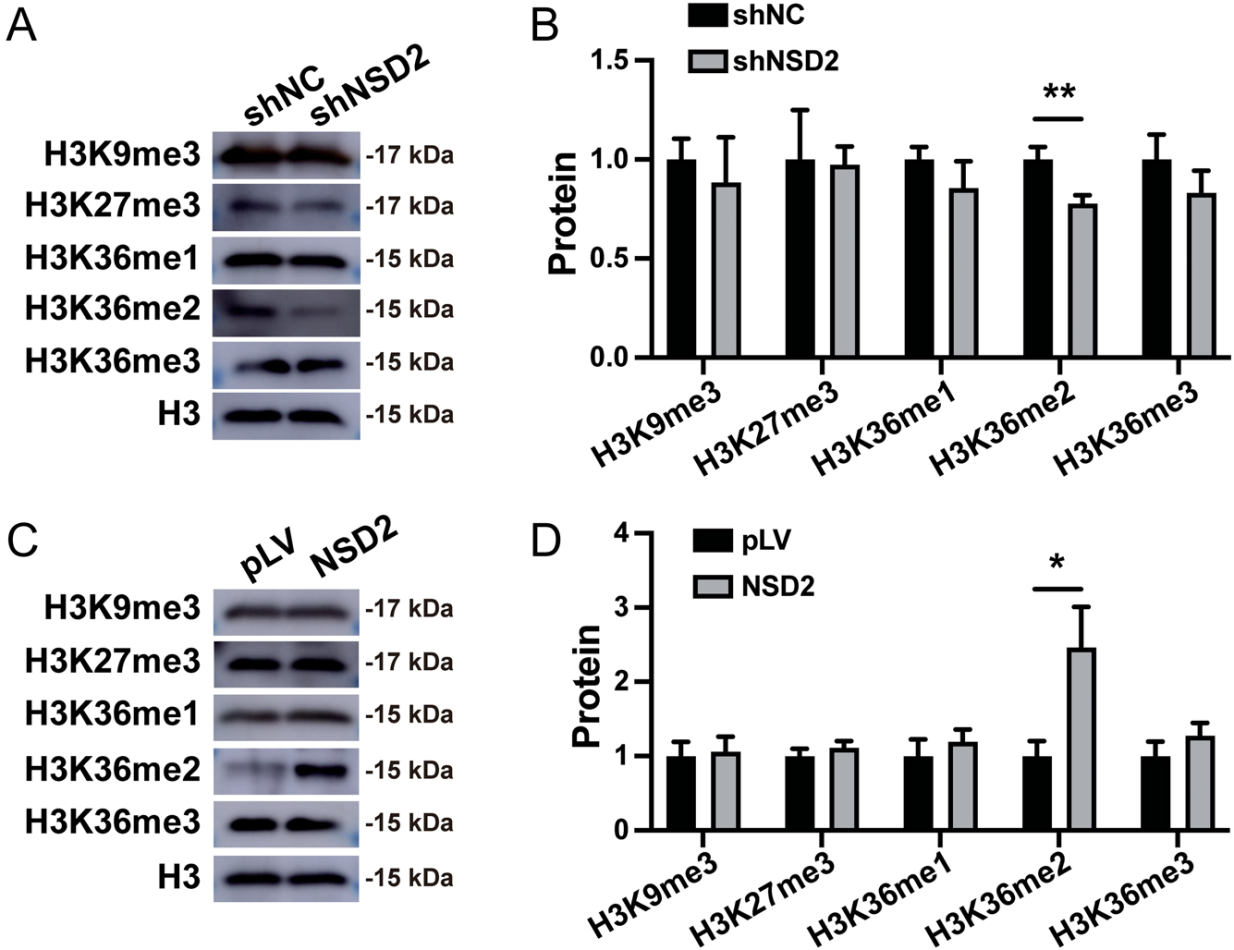
NSD2 mainly regulates the enrichment of H3K36me2 in HESCs. (A, B) Downregulation of NSD2 leads to decreased H3K36me2 levels. (C, D) Upregulation of NSD2 leads to increased H3K36me2 levels. Each set of experiments was replicated at least three times. shNC, pLV-Neo-U6; shNSD2, pLV[shRNA]-Neo-U6>hNSD2; pLV, pLV-Neo-CMV; NSD2, pLV [Exp]-Neo-CMV>hNSD2; *P < 0.05; **P < 0.01).

Histone methylation regulates the cell cycle (Van Rechem *et al.* 2021). We detected H3K9me3, H3K27me3, H3K36me1, H3K36me2, and H3K36me3 after *NSD2* knockdown or overexpression. Western blotting showed that after knockdown or overexpression of *NSD2*, only H3K36me2 levels showed the same expression tendency as NSD2, and the differences were significant (Fig. 3A, B, C, and D).

### H3K36me2 levels are reduced in the endometrial stromal cell of patients with RIF

**Figure 4:**
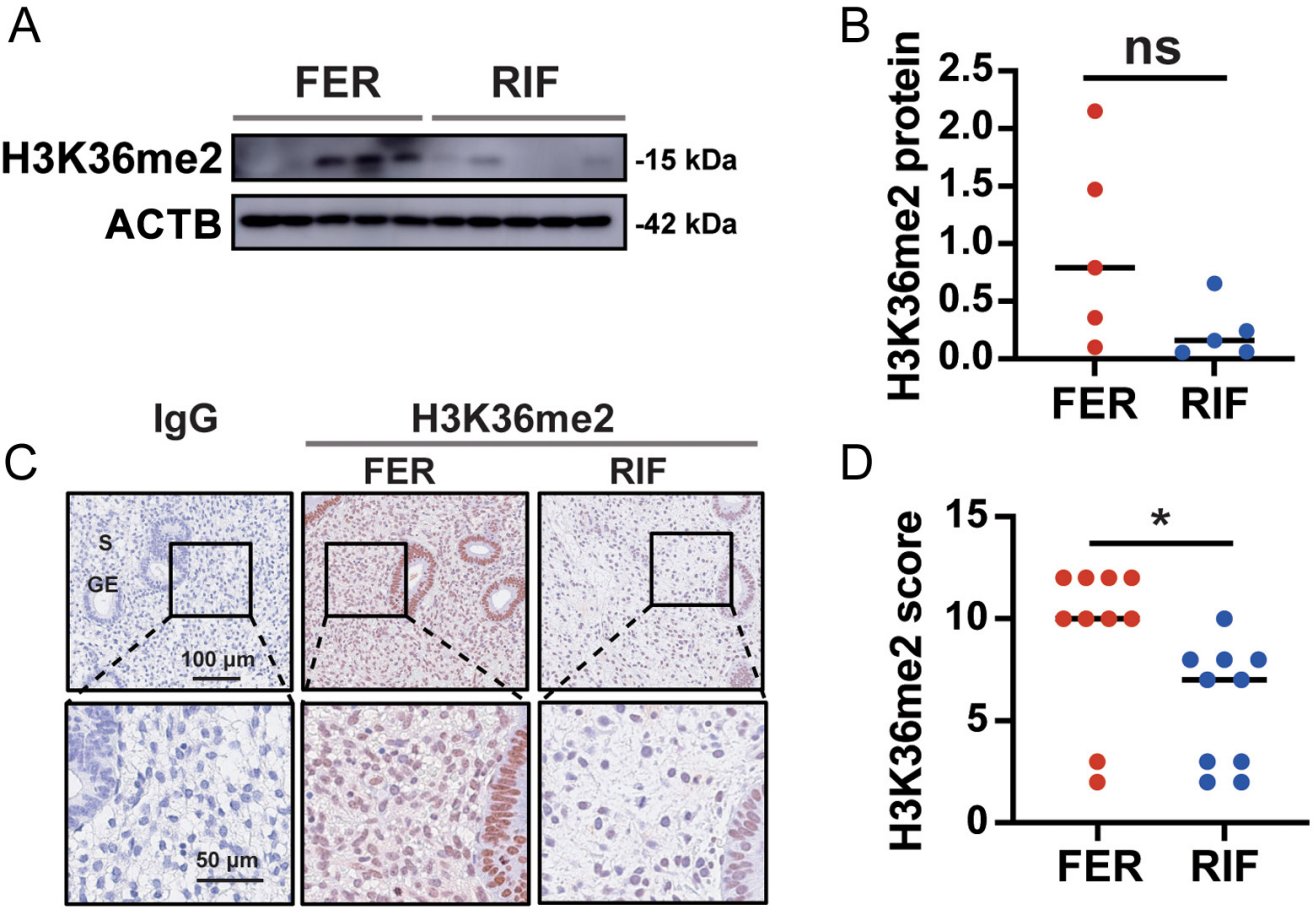
Reduced H3K36me2 levels in the proliferative endometrium of patients with RIF compared with those in the FER group. (A, B) Western blot showing H3K36me2 protein levels in the proliferative endometrium of patients with RIF (*n* = 5) and FER controls (*n* = 5). (C, D) Representative immunohistochemical images depicting the expression of NSD2 in the endometrium from patients with RIF (*n* = 10) and FER controls (*n* = 10). The negative control was normal rabbit IgG. GE, glandular epithelium; S, stroma; ns: not statistically different; *P < 0.05.

Western blotting showed a nonsignificant trend toward reduced H3K36me2 levels in the proliferative-phase endometrium of the RIF group compared with that of the FER group (*P* = 0.0785; Fig. 4A and B). Immunohistochemical staining showed that H3K36me2 levels in the endometrial stromal cells from patients with RIF were significantly reduced (Fig. 4C and D). Accordingly, H3K36me2 levels in the endometrial stromal cell of patients with RIF were decreased.

### *NSD2* knockdown blocks the cell cycle of HESCs

**Figure 5:**
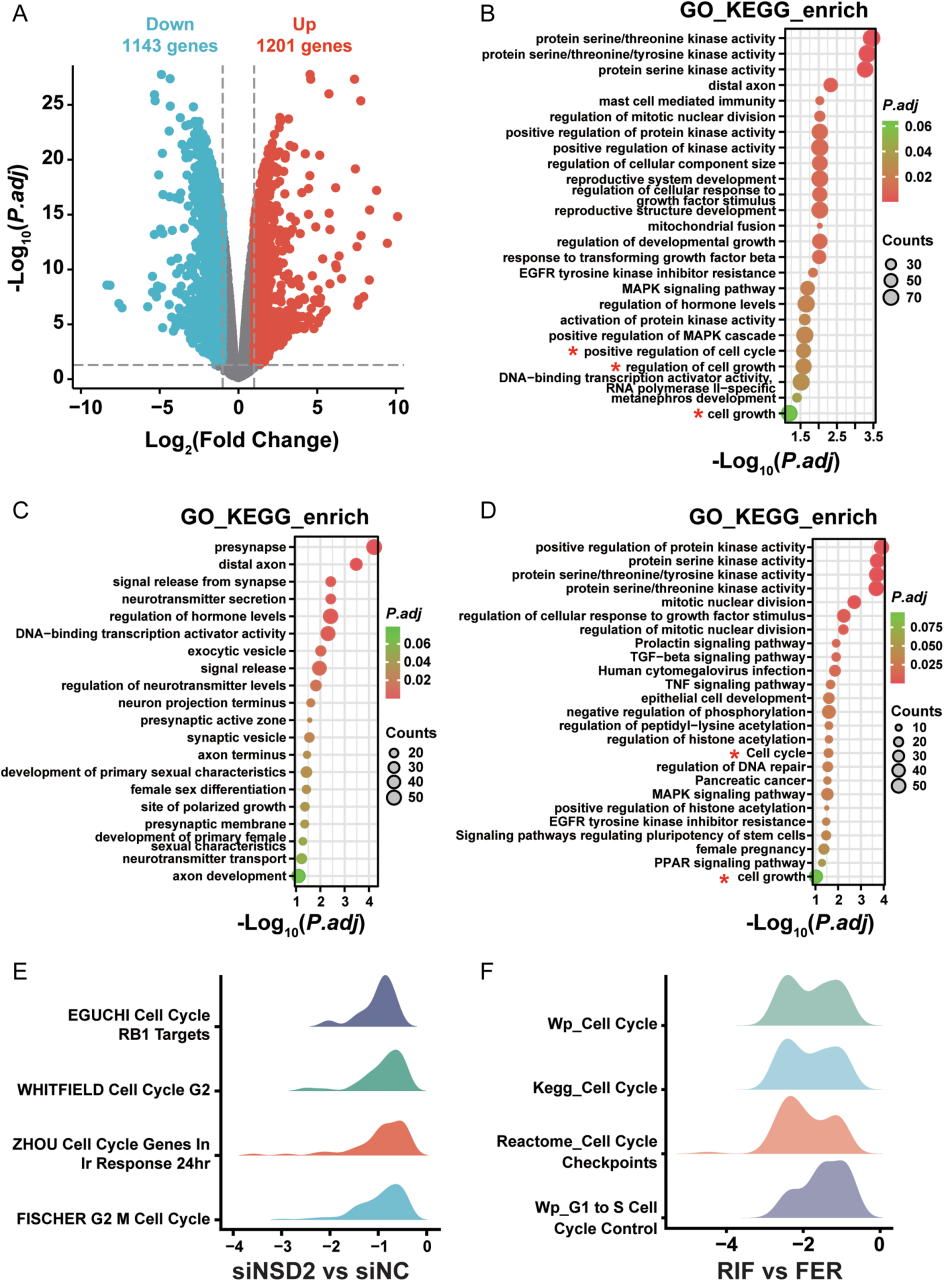
*NSD2* knockdown in HESCs leads to downregulated cell cycle-related gene sets. (A) Volcano plot showing differentially expressed genes after knockdown of *NSD2*. (B) Enriched GO terms and KEGG pathways of DEGs between the siNSD2 group and the siNC group. (C) Enriched GO terms and KEGG pathways of upregulated DEGs between the siNSD2 group and the siNC group. (D) Enriched GO terms and KEGG pathways of downregulated DEGs between the siNSD2 group and the siNC group. (E) GSEA showing genes enriched by *NSD2* knockdown in HESCs. (F) GSEA showing genes enriched between patients with RIF and the FER group.

RNA-seq showed the differentially expressed genes (DEGs) between the siNSD2 group and the siNC group. Knockdown of *NSD2* resulted in 1143 downregulated DEGs and 1201 upregulated DEGs (Fig. 5A). GO and KEGG analyses showed that the DEGs were involved in cell growth and cell cycle-related gene sets (Fig. 5B). This phenomenon was also observed in the downregulated genes between the siNSD2 group and siNC group (Fig. 5D), but not observed for the upregulated genes (Fig. 5C). GSEA of the DEGs revealed that enrichment in the EGUCHI Cell Cycle RB1 Targets, WHITFIELD Cell Cycle G2, Zhou_Cell Cycle Genes in Ir Response 24hr, and FISCHER G2 M Cell Cycle was all downregulated after *NSD2* knockdown (Fig. 5E). The expression pattern of cell cycle-related gene sets was similar between the proliferative-phase endometrium of patients with RIF and the FER controls. The genes involved in Wp_Cell Cycle, Kegg_Cell Cycle, Reactome_Cell Cycle Checkpoints, and Wp_G1 to S Cell Cycle Control were mainly downregulated in patients with RIF compared with those in the FER controls (Fig. 5F). These results suggest NSD2 reduction probably blocks the cell cycle of endometrial stromal cells in patients with RIF.

### *NSD2* knockdown reduces H3K36me2 on the *MCM7* promoter

**Figure 6:**
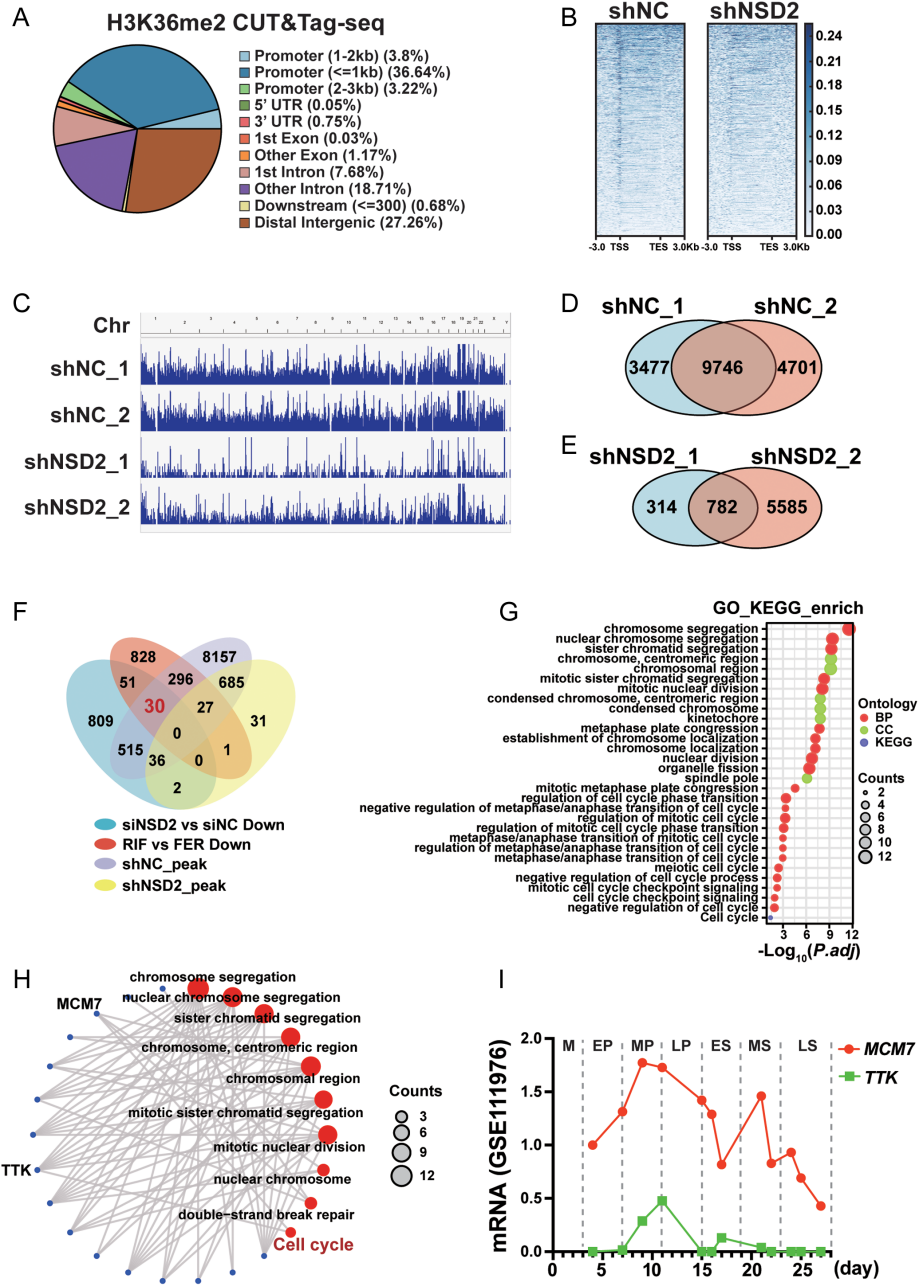
*NSD2* knockdown in HESCs leads to reduced H3K36me2 modifications and the corresponding functional changes. (A) Pie chart depicting the genomic distribution of H3K36me2 peaks. (B) Heatmap showing the occupancy for H3K36me2 across the genome in shNSD2 cells and shNC cells. (C) Chromosomal views showing broadly reduced H3K36me2 peaks after* NSD2* knockdown. (D) Venn diagram illustrating the overlap of CUT&Tag-Seq peaks in shNC cells. (E) Venn diagram illustrating the overlap of CUT&Tag-Seq peaks in shNSD2 cells. (F) Venn diagram of downregulated genes after knockdown of *NSD2* and decreased genes in patients with RIF and the common peaks in (D), (E). (G) GO terms and KEGG pathways for genes in the overlap of genes downregulated after *NSD2* knockdown, genes decreased in patients with RIF, and H3K36me2 peaks in shNC cells but not in shNSD2 cells. (H) Connection between genes, GO terms, and Kegg_Cell Cycle. (I) *MCM7* and* TTK* mRNA levels in endometrial stromal cells during the menstrual cycle (GES111976). The details of the staging of the menstrual cycle are described in Supplementary Table 3. M, menstrual phase; EP, early-proliferative; ≤MP, mid-proliferative; LP, late-proliferative; ES, early-secretory; MS, mid-secretory; LS, late-secretory.

**Figure 7:**
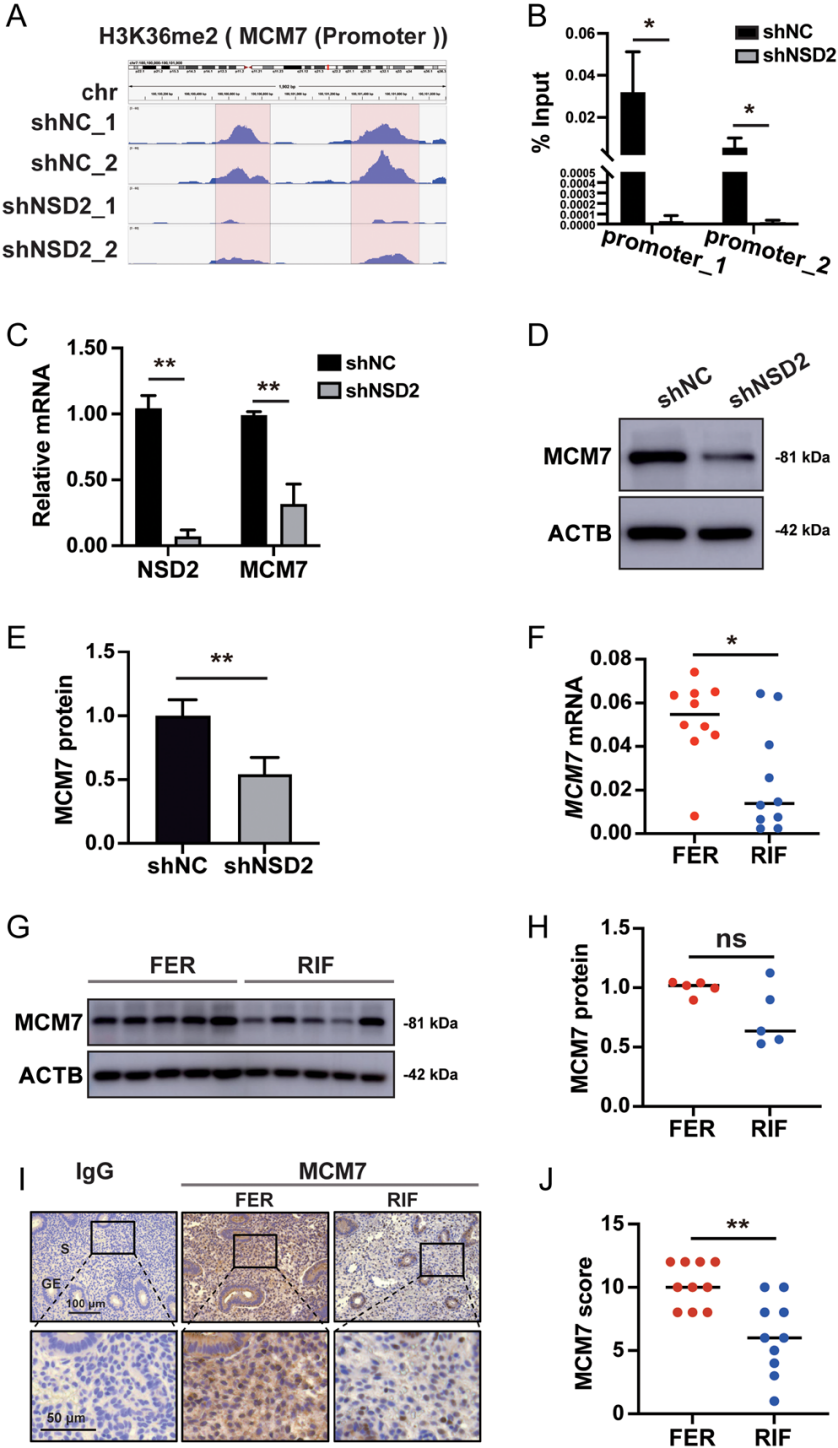
NSD2 regulates *MCM7* expression through H3K36me2 modifications on promoter regions. (A) IGV presentation of the enrichment of H3K36me2 in shNC and shNSD2 cells on *MCM7* promoter regions. (B) ChIP-qPCR validation of the CUT&Tag-Seq in (A). (C) *MCM7* is downregulated by knockdown of *NSD2*. (D, E) Western blot showing that knockdown *NSD2* reduces MCM7 protein levels. (F) *MCM7* expression decreased in the proliferative endometrium of patients with RIF (*n* = 10) compared with FER controls (*n* = 10). (G, H) Protein levels of MCM7 in the proliferative endometrium of patients with RIF (*n* = 5) and FER controls (*n* = 5). (I, J) Representative immunohistochemical images depicting the expression of MCM7 in the endometrium from patients with RIF (*n* = 10) and FER controls (*n* = 10). The negative control was normal rabbit IgG. GE, glandular epithelium; S, stroma; ns: not statistically different; *P < 0.05; **P < 0.01.

H3K36me2 was broadly distributed over gene areas (Fig. 6A) and shNSD2 HESCs exhibited reduced H3K36me2 levels (Fig. 6B and C). Overlap of CUT&Tag-Seq peaks in shNC HESCs (Fig. 6D) and shNSD2 HESCs (Fig. 6E) showed that *NSD2* knockdown of NSD2 reduced the number of H3K36me2 common peaks from 9746 to 782. The overlap between genes downregulated in siNSD2 HESCs, genes reduced in patients with RIF, and shNC HESCs peaks but not shNSD2 HESCs peaks, showed that NSD2 mainly reduced the H3K36me2 domains on 30 genes to regulate HESC proliferation (Fig. 6F). GO and KEGG analyses showed that these genes were mainly associated with chromosome organization and cell cycle (Fig. 6G). Cell cycle-related genes *MCM7* and *TTK* (encoding TTK protein kinase) are associated with chromatin-related functions (Fig. 6H). Published single-cell data (GSE111976) showed that *MCM7* and *TTK* expression levels gradually increase in the early- and mid-proliferative-phase endometrium. However, *TTK* expression was too low in the dataset (Fig. 6I). Thus, we hypothesized that NSD2 functions in HESCs proliferation by transcriptionally regulating *MCM7*. Integrative Genomics Viewer (IGV) showed a markedly lower H3K36me2 level in shNSD2 HESCs on the *MCM7* promoter (Fig. 7A), which was validated using ChIP-qPCR (Fig. 7B). qRT-PCR (Fig. 7C) and western blotting (Fig. 7D and E) showed that *NSD2* knockdown significantly reduced MCM7 expression. These data suggested that NSD2 downregulates proliferation marker MCM7 by reducing H3K36me2 levels on its promoter.

### MCM7 is reduced in the proliferative-phase endometrium of patients with RIF

qRT-PCR showed that *MCM7* mRNA expression was significantly reduced in the proliferative-phase endometrium of patients with RIF (Fig. 7F). The protein level of MCM7 was decreased in the patients with RIF, but not significantly (*P* = 0.2222) (Fig. 7G and H). Immunohistochemistry further confirmed that MCM7 was decreased in the proliferative-phase endometrium (Fig. 7I and J).

## Discussion

**Figure 8:**
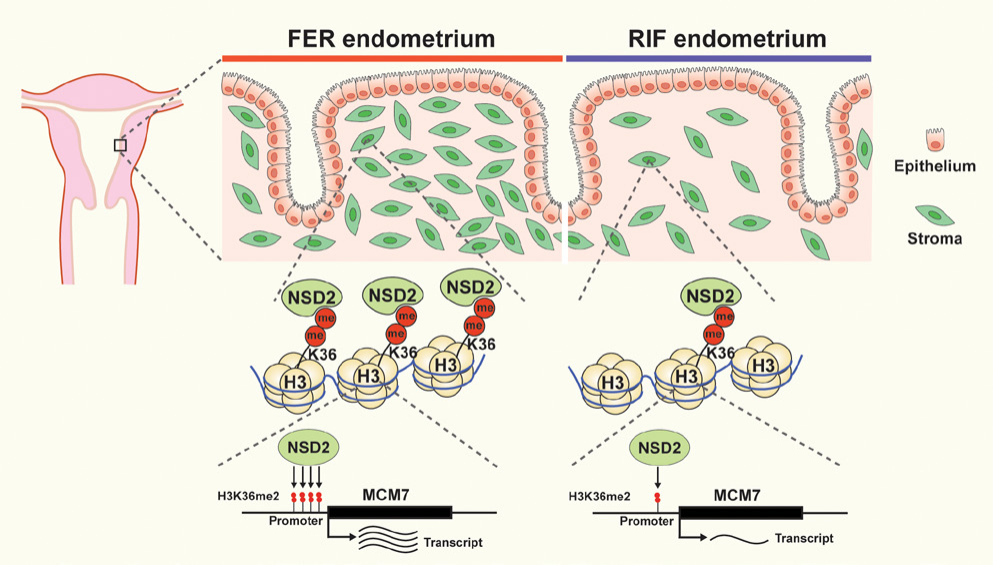
Working model showing how decreased NSD2 impairs the proliferation of endometrial stromal cells in patients with RIF.

The present study confirmed that histone methyltransferase NSD2 plays an important role in regulating endometrial stromal cell proliferation via dimethylating H3K36. Furthermore, the genome-wide H3K36me2 profile revealed that inhibition of NSD2 reduced global H3K36me2 binding regions on chromatin in HESCs. CUT&Tag-seq and RNA-seq identified that NSD2 promoted MCM7 expression in HESCs via the regulation of the H3K36me2 level at the promoter of *MCM7*. Moreover, NSD2 and MCM7 expression are decreased in the proliferative-phase endometrium of RIF. Therefore, our data clarified a novel epigenetic regulatory function of the NSD2-H3K36me2-MCM7 pathway in promoting endometrial stromal cell proliferation, implying a potential role for NSD2 in the pathogenesis of RIF (Fig. 8).

Endometrial stromal cells play an important role in repairing the endometrium after menstrual bleeding (Wang *et al.* 2020*a*). Endometrial stromal cell proliferation and differentiation are crucial to decidualization, thereby ensuring the implantation of the embryo (Yuan *et al.* 2019). A study found that decreased endometrial proliferation might lead to poor pregnancy rates (Cetin *et al.* 2021). Therefore, to investigate the pathogenic mechanism of RIF, it is important to compare the gene expression profiles between the RIF group with the FER group. Previous studies have reported that impaired endometrial proliferation might lead to pathological changes consistent with RIF (Zhang *et al.* 2018, Wang *et al.* 2019, Zhou *et al.* 2022). Recently, epigenetic aberrations have been implicated in endometrial function. EZH2 was reported to regulate endometrial proliferation by reprogramming global H3K27me3 levels (Mesa *et al.* 2020). Moreover, Liu *et al.* found that deletion of Men1 reduced the levels of H3K4me3, resulting in poor decidualization and embryo implantation failure (Liu *et al.* 2022). However, more study is required to unravel the role of epigenetic modifications in the endometrial proliferation in RIF. NSD2 is implicated in diverse diseases as the predominant histone methyltransferase catalyzing H3K36me2 (Lam *et al.* 2022). During tumor formation, high NSD2 expression catalyzes H3K36me2 at nuclear factor kappa B (NF-kB) target gene promoters to activate the NF-kB pathway, thereby promoting tumor growth (Yang *et al.* 2012). Meanwhile, Zhao *et al.* reported that in primary colon cancer cells, *NSD2* silencing or knockout significantly downregulated H3K36me2, the expression of multiple oncogenes, and protein kinase B (AKT) activation, thereby inhibiting cell cycle progression, proliferation, migration, and invasion (Zhao *et al.* 2021). In the present study, we found that *EZH2* and *NSD2* were significantly decreased in the proliferative-phase endometrium of patients with RIF compared with those in the FER controls. The mechanism by which EZH2 regulates endometrial function is relatively clear (Grimaldi *et al.* 2011, Fukui *et al.* 2023, Sirohi *et al.* 2023), while the mechanism by which NSD2 regulates endometrial function is still unclear. Therefore, we chose NSD2 for further research. Then, evidence, including CUT&Tag-seq, RNA-seq, ChIP-qPCR, *in vitro* cell proliferation assays, and analyses of human RIF specimens, supports the role of NSD2 in mediating the proliferation of endometrial stromal cells via reprograming H3K36me2 at the promoter of *MCM7*. However, there are still limitations in our study. We adopted a lower threshold in the tolerable range rather than a strict threshold during the differential gene expression analysis of tissue RNA-seq data. Estrogen was not added directly to the cell medium as a variable for cell proliferation. The sample sizes in this study are small and a non-stringent definition of RIF was selected in our study.

Reduced proliferation rates of endometrial stromal cells might disrupt decidualization and embryo implantation (Woods *et al.* 2017). During embryo implantation failure, however, the endometrium possesses an abnormal proliferative property that contributes to pathogenesis (Taylor & Fei 2005, Sekulovski *et al.* 2021). In the rat uterus, MCM7 expression was maximal at the proestrous stage, as confirmed by increased estradiol induction of endometrial cell proliferation (Dery *et al.* 2003). In this study, *MCM7* and *TTK* were identified as the major downstream cell cycle-related genes. The re-analysis of published single-cell RNA-seq data and the protein data from the Human Protein Atlas Database (https://www.proteinatlas.org/) similarly suggested MCM7 was expressed at medium levels, whereas TTK was low or not detected in endometrial stroma. So, MCM7 was selected as the candidate gene. MCM7 is a crucial subunit of the minichromosome maintenance complex, which exerts the critical first step in DNA helicase-mediated unraveling of duplex DNA at the DNA replication fork, thereby determining the DNA synthesis rate (Hanahan & Weinberg 2000, Murayama *et al.* 2018). MCM7 expression is increased in a variety of tumors, including hepatocellular, neuroblastoma, hypopharyngeal, prostate, and cervical carcinomas, acting as a marker for cell proliferation (Honeycutt *et al.* 2006, Qu *et al.* 2017). Herein, *NSD2* knockdown reduced the H3K36me2 level on the* MCM7* promoter region and *MCM7* expression was downregulated after NSD2 knockdown. ChIP-qPCR confirmed the CUT&Tag-Seq results that loss of NSD2 decreased H3K36me2 on the promoter of *MCM7*. These results were consistent with a previous report that NSD2-mediated deposition of H3K36me2 activates genes (Brien *et al.* 2016). In addition, consistent with the level of NSD2 and H3K36me2, the MCM7 level was reduced in the proliferative-phase endometrium of patients with RIF.

In conclusion, by comparing human and cellular experimental data related to HESC proliferation, this study supplies new insights on RIF pathogenesis by exploring the function of NSD2, which was shown to regulate the proliferation of endometrial stromal cells by changing the enrichment of H3K36me2 at the promoter region of target genes. Among these genes, *MCM7* was identified to be strictly regulated by NSD2 *in vitro*. To determine the interaction of endometrial stromal cells with other endometrial cell types (e.g. endometrial epithelial cells) during the proliferative phase, further studies (e.g. primary cell cultures, endometrial organoid cultures, single-cell sequencing, and *in vivo* experiments) are still required. Our study will help to understand the mechanism of endometrial dysfunctions underlying embryo implantation failure. Furthermore, these results can provide guidance to predict embryo implantation failure, and the NSD2-H3K36me2-MCM7 pathway might have potential as a therapeutic target to treat RIF.

## Supplementary materials

Supplemental Figure 1. Efficiency of the knockout and overexpression of NSD2. (A) qRT-PCR showing NSD2 mRNA levels in HESCs transfected with shNC or shNSD2. (B, C) Western blot showing NSD2 levels in HESCs transfected with shNC or shNSD2. (D) qRT-PCR showing NSD2 mRNA levels in HESCs transfected with pLV or NSD2. (E, F) Western blot showing NSD2 levels in HESCs transfected with pLV or NSD2. (shNC: pLV-Neo-U6; shNSD2: pLV[shRNA]-Neo-U6 > hNSD2; pLV: pLV-Neo-CMV; NSD2: pLV[Exp]-Neo-CMV > hNSD2; *p < 0.05; **p < 0.01).

Supplemental Figure 2. RNA-sequencing reveals transcriptional changes in patients with RIF compared with the FER group. (A) Volcano plots showing the differentially expressed genes between the proliferative endometrium of the RIF and FER groups. (B) EZH2 and NSD2 expression levels are decreased in patients with RIF. (C) GO terms and KEGG pathways between the RIF and FER groups.

Supplemental Figure 3. Immunofluorescence staining of NSD2 in HESCs.

Supplemental Figure 4. Uncropped images of western blots.

Supplementary Material

Supplementary Tables

## Declaration of interest

The authors declare that there is no conflict of interest that could be perceived as prejudicing the impartiality of the study reported.

## Funding

This work was supported by the National Key Research and Development Program of Chinahttp://dx.doi.org/10.13039/501100012166 (2018YFC1002800), the Innovative Research Team of High-level Local Universities in Shanghai (SHSMU-ZLCX20210202), the National Natural Science Foundation of Chinahttp://dx.doi.org/10.13039/501100001809 (81971403, 82171669), the Shanghai Jiao Tong Universityhttp://dx.doi.org/10.13039/501100004921 Trans-Med Awards Research (20210201), and the Funds for Outstanding Newcomers, Shanghai Sixth People's Hospital (X-3664).

## Ethical approval and consent to participate

The study protocol was approved by the Medical Ethics Committee of the International Peace Maternity & Child Health Hospital of China Welfare Institute, Shanghai ((GKLW) 2021-49).

## Patient consent

Written informed consent was obtained from all the participants before enrolment (FER and RIF groups).

## Data availability

The raw data can be provided upon request without undue reservation. The sequencing data reported in this paper are deposited at NCBI SRA: PRJNA922202, PRJNA922059, and PRJNA922217.

## Author contribution statement

YL designed the study. CMQ and XWW performed major parts of the experiments. JYW and XQL performed a part of the experiments. CMQ and XWW wrote the manuscript. All authors contributed to editing the manuscript and approved the final version of the manuscript.
