# Supplementary figures and images for: Decreased NSD2 impairs stromal cell proliferation in human endometrium via reprogramming H3K36me2
Source: Reproduction. 2024 Feb 12;167(3):e230254. doi: 10.1530/REP-23-0254 (PMC10895284; doi:10.1530/REP-23-0254)

# Supplemental Figure 1

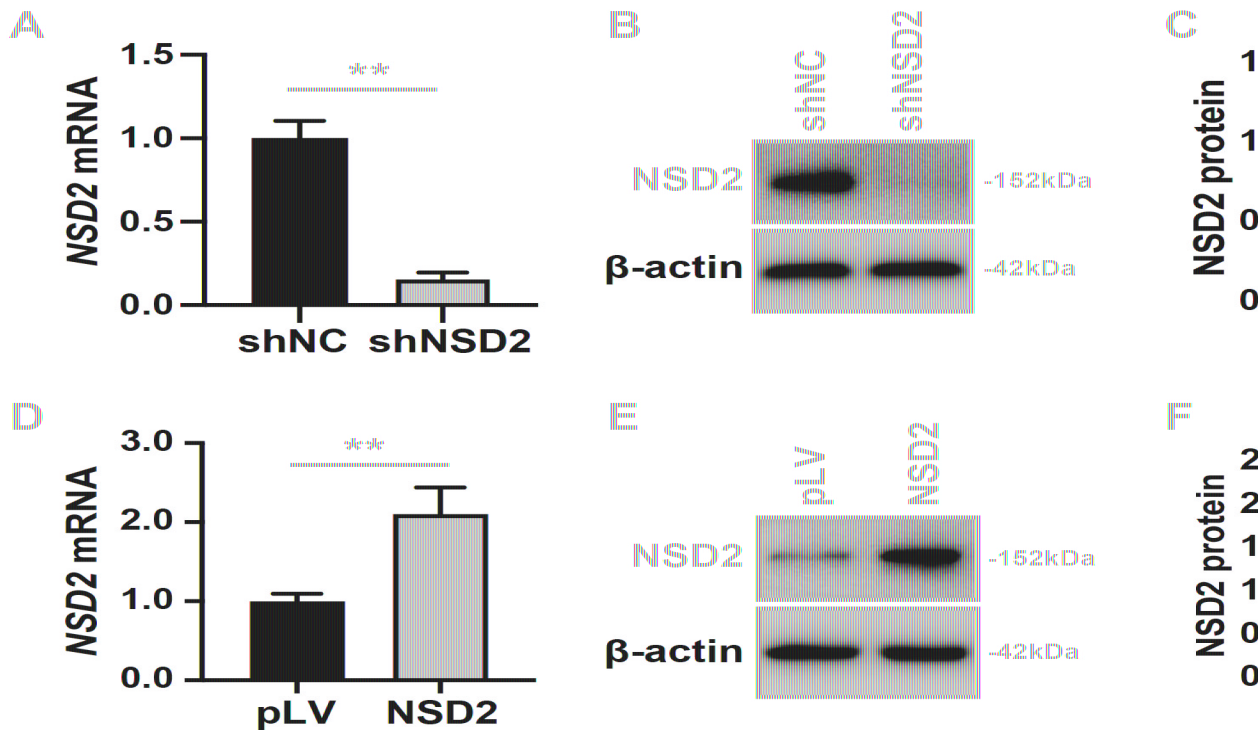

Supplement: Supplemental Figure 1. Efficiency of the knockout and overexpression of NSD2. (A) qRT-PCR showing NSD2 mRNA levels in HESCs transfected with shNC or shNSD2. (B, C) Western blot showing NSD2 levels in HESCs transfected with shNC or shNSD2. (D) qRT-PCR showing NSD2 mRNA levels in HESCs transfected wit [file supplementary_figure_1.pdf]

Supplemental Figure 2

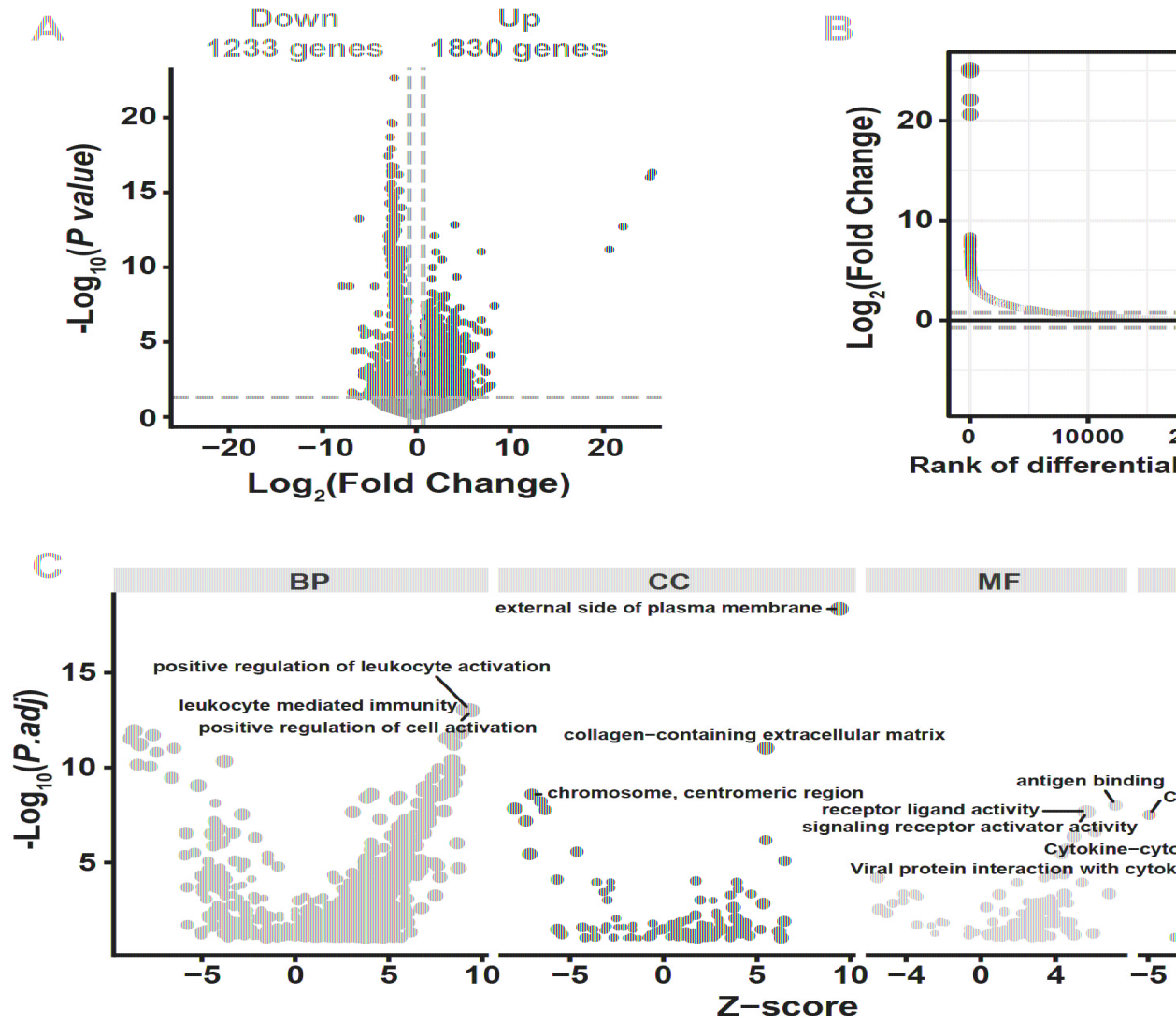

Supplement: Supplemental Figure 2. RNA-sequencing reveals transcriptional changes in patients with RIF compared with the FER group. (A) Volcano plots showing the differentially expressed genes between the proliferative endometrium of the RIF and FER groups. (B) EZH2 and NSD2 expression levels are decreased in p [file supplementary_figure_2.pdf]

## Supplemental Figure 3

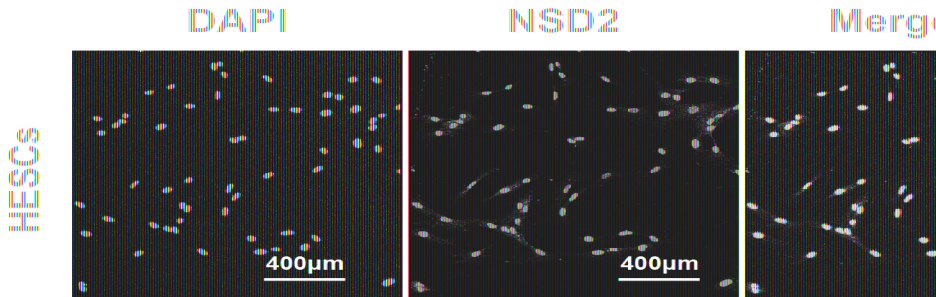

Supplement: Supplemental Figure 3. Immunofluorescence staining of NSD2 in HESCs. [file supplementary_figure_3.pdf]
