# Supplementary material for: Decreased NSD2 impairs stromal cell proliferation in human endometrium via reprogramming H3K36me2
Source: Reproduction. 2024 Feb 12;167(3):e230254. doi: 10.1530/REP-23-0254 (PMC10895284; doi:10.1530/REP-23-0254)

Supplemental Figure 4

Figure 1D

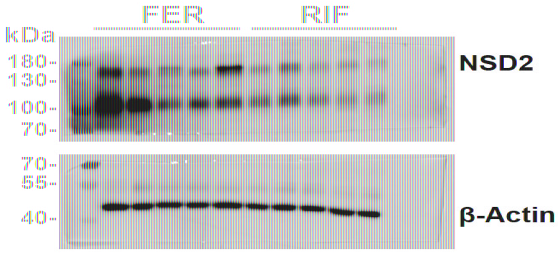

Figure 3A

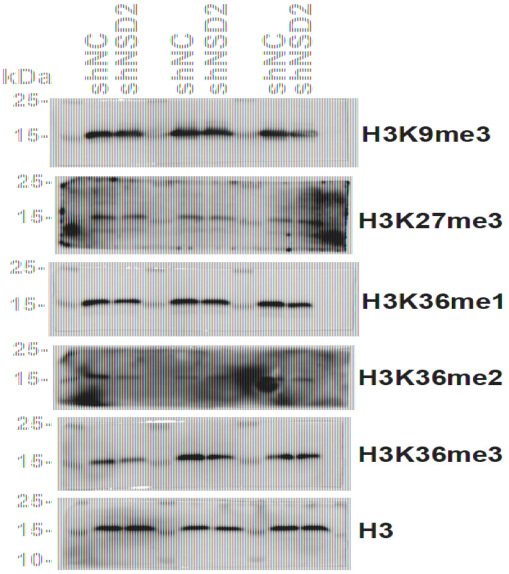

Figure

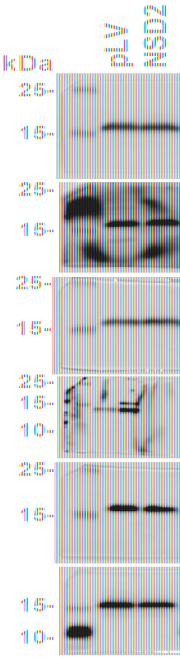

Figure 4A

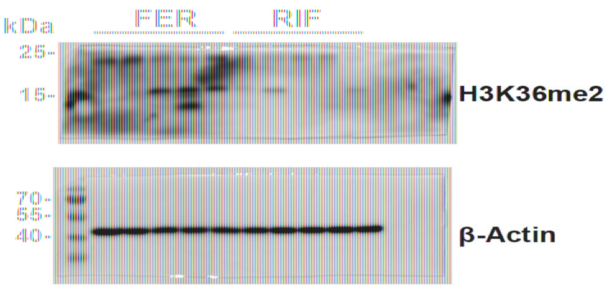

Figure 7D

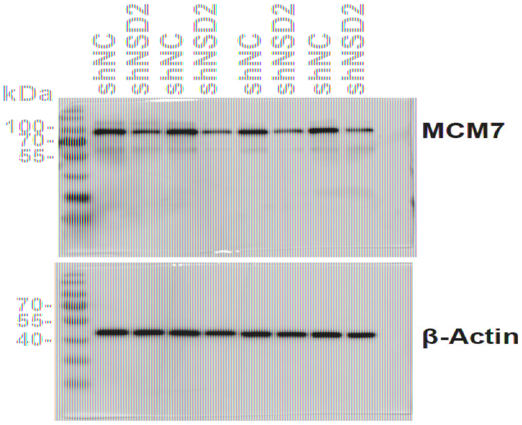

Supplemental Figure

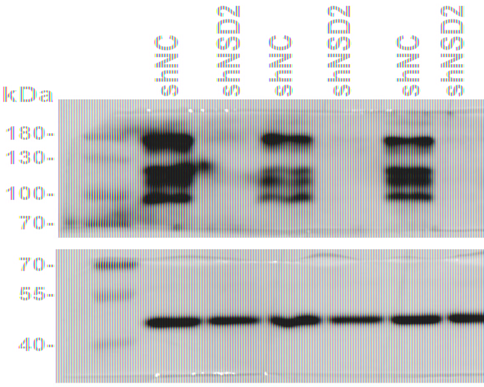

Figure 7G

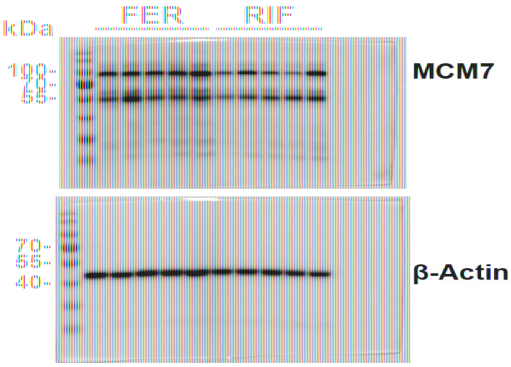

Supplemental Figure

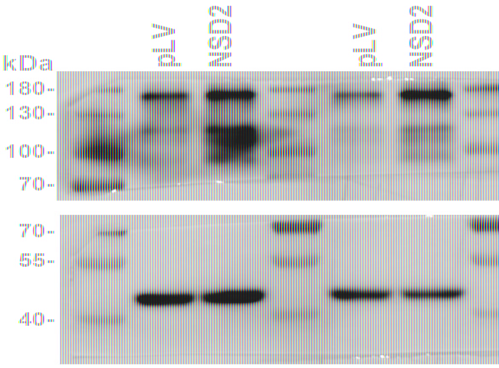

Supplement: Supplemental Figure 4. Uncropped images of western blots. [file supplementary_figure_4.pdf]
