# Supplementary material for: Decreased NSD2 impairs stromal cell proliferation in human endometrium via reprogramming H3K36me2
Source: Reproduction. 2024 Feb 12;167(3):e230254. doi: 10.1530/REP-23-0254 (PMC10895284; doi:10.1530/REP-23-0254)
Supplement: Supplementary Material [file supplementary_material.pdf]

Supplemental Methods

Immunofluorescence

For immunofluorescence, HESCs were seeded in 24-well plates one day before the experiment. The next day, at a confluence of 30%, the cells were then fixed using 4% paraformaldehyde for 20 min at room temperature. After washing with PBS, the cells were then permeabilized using 0.3% Triton X-100 in PBS. Subsequently, the cells were blocked using 5% BSA in PBS for 1 h at room temperature and then incubated with primary antibodies against NSD2 (1:250, ab223694, Abcam, Cambridge, UK) overnight at 4 °C. Next day, after washing with PBS three times, the cells were incubated with Alex Fluor 488-conjugated goat anti-Rabbit IgG (Life Technologies, Carlsbad, CA, USA). Finally, cell nuclei were counterstained with 4,6-diamino-2-phenylindole (DAPI) (1 μg/ml, Abcam) and cells were observed under a fluorescence microscope (Leica DMi8 microscope, Leica Microsystems, Wetzlar, Germany).

Secondary data analysis

To investigate gene expression patterns throughout the female menstrual cycle, we analyzed published single-cell RNA-seq data of the human endometrium during the menstrual cycle (Wang et al., 2020). The processed single cell endometrial dataset (GSE111976) was downloaded from the NCBI GEO database and filtered and analyzed using the R toolkit Seurat (Stuart et al., 2019). Single-cell data were filtered using the following exclusion criteria: 1) genes covered by less than 3 cells, 2) cells

expressing less than 200 genes, 3) cells with more than 5% of mitochondrial genes, and 4) cells with more than 7500 genes (Tan et al., 2023). Then, we selected out the labeled stromal fibroblasts and calculated the mean gene expression values at each time point and plotted them using GraphPad Prism software version 8 (GraphPad Inc, La Jolla, CA, USA).

Immunohistochemical scoring

Immunohistochemical scoring was based on the levels and areas of gene expression and was performed in a double-blinded manner by the clinical pathologists of the International Peace Maternity & Child Health Hospital. Score = ∑ (Pi × i), where i = intensity of staining with a value of 0, 1, 2 or 3 (negative, weak, moderate, or strong, respectively) and Pi is the percentage of stained endometrial cell for each intensity with a value of 0, 1, 2, 3 or 4 (less than 5%, 5% to 25%, 26% to 50%, 51% to 75%, greater than 75%, respectively) (Zhao et al., 2019, Liu et al., 2020).

Statistical analyses of qRT-PCR data

The comparative threshold cycle (Ct) method was used to analyze the qRT-PCR data (Schmittgen and Livak, 2008). ΔCt was calculated by subtracting the Ct value of *ACTB* from the target gene Ct value. ΔΔCT were obtained by subtracting the ΔCT of the reference point from the ΔCT of the experimental group target gene (Xiao et al., 2017). For HESCs cells, the $2^{-\Delta\Delta Ct}$ method was used to analyze the relative mRNA expression (Livak and Schmittgen, 2001). There is no means to justify RIF sample

with FER samples; therefore, $2^{-\Delta Ct}$ method was used for human endometrial tissue samples (Schmittgen and Livak, 2008).

Statistical analysis

The Shapiro-Wilk test was used to determine normality. For data not conforming to a normal distribution, differences in means were tested for statistical significance using a two-tailed Mann–Whitney test (Figure 1G, 4D, 7F, 7G). Data are presented as the median (first and third quartile). For data that passed the normality test, Levene’s test of homogeneity of variance was further performed. For normally distributed samples with equal variance, an unpaired two-tailed Student’s t-test was used for the comparison between two groups (Figure 1C, 1E, 2F, 2L, 3B, 3D, and Supplemental Figure 1 A, 1C, 1D, 1F). For normally distributed samples with unequal variance, an unpaired two-sided Welch’s t-test was used for the comparison between two groups (Figure 4B, 7H). Comparisons between multiple groups were performed using two-way ANOVA for data with normal distribution and equal variance (Figure 2C, 2D, 2I, 2J). Data are shown as the mean ± standard deviation (SD). All *P*-values are two-sided and a value less than 0.05 was regarded as statistically significant. All statistical calculations were performed using SPSS 26 (IBM Corp., Armonk, NY, USA).

References

Liu XY, Jiang W, Ma D, Ge LP, Yang YS, Gou ZC, Xu XE, Shao ZM & Jiang YZ 2020 SYTL4 downregulates microtubule stability and confers paclitaxel resistance in triple-negative breast cancer. *Theranostics* **10** 10940-10956. (https://doi.org/10.7150/thno.45207)

Livak KJ & Schmittgen TD 2001 Analysis of relative gene expression data using real-time quantitative PCR and the 2(-Delta Delta C(T)) Method. *Methods (San Diego, Calif.)* **25** 402-8. (https://doi.org/10.1006/meth.2001.1262)

Schmittgen TD & Livak KJ 2008 Analyzing real-time PCR data by the comparative C(T) method. *Nature protocols* **3** 1101-8. (https://doi.org/10.1038/nprot.2008.73)

Xiao E, Mattos M, Vieira GHA, Chen S, Corrêa JD, Wu Y, Albiero ML, Bittinger K & Graves DT 2017 Diabetes Enhances IL-17 Expression and Alters the Oral Microbiome to Increase Its Pathogenicity. *Cell Host Microbe* **22** 120-128 e4. (https://doi.org/10.1016/j.chom.2017.06.014)

Zhao S, Liu XY, Jin X, Ma D, Xiao Y, Shao ZM & Jiang YZ 2019 Molecular portraits and trastuzumab responsiveness of estrogen receptor-positive, progesterone receptor-positive, and HER2-positive breast cancer. *Theranostics* **9** 4935-4945. (https://doi.org/10.7150/thno.35730)
