# Supplementary material for: Decreased NSD2 impairs stromal cell proliferation in human endometrium via reprogramming H3K36me2
Source: Reproduction. 2024 Feb 12;167(3):e230254. doi: 10.1530/REP-23-0254 (PMC10895284; doi:10.1530/REP-23-0254)
Supplement: Supplementary Tables [file supplementary_tables.pdf]

Supplemental Table 1. The sequences of siNSD2

siRNA	Sense 5′ to 3′	Antisense 5′ to 3′
NSD2	GGGUCAUGCAGAAGUUUAATT	UUAAACUUCUGCAUGACCCTT

Supplemental Table 2. Primers used in the present study

Primer name	Primer sequence (5′ to 3′)
	Gene primers
NSD2-qPCR-F	TGTGTGAGCTGCCATGCTTCCA
NSD2-qPCR-R	TGAGCATCCTGCTGCCAGACAA
MCM7-qPCR-F	CACAGCCATCCACGAGGTCAT
MCM7-qPCR-R	AGAGCAGTGCAGCAGGTAGC
β-Actin-qPCR-F	CACCATTGGCAATGAGCGGTTC
β-Actin-qPCR-R	AGGTCTTTGCGGATGTCCACGT
	ChIP primers
MCM7 (1)-ChIP-F	AATCATGGCCGTTCTGGAGAGT
MCM7 (1)-ChIP-R	GGCGGAGGAGAATCGCTCTTAA
MCM7 (2)-ChIP-F	TGGAGGGAGTGTCGTGTAAACA
MCM7 (2)-ChIP-R	GGCCATCACACCCAGAGACA

Supplemental Table 3. Cycle phases in relation to the days of the menstrual cycle

Cycle phase	Days of cycle
Menstrual phase (M)	1–3
Early-proliferative (EP)	4–7
Mid-proliferative (MP)	8–11
Late-proliferative (LP)	12–15
Early-secretory (ES)	16–19
Mid-secretory (MS)	20–23
Late-secretory (LS)	24–28

Note: The menstrual cycle is 28 days in length.
